# High-resolution dataset (2017-2023) of physical-geographical predictors for machine learning modelling of fluvial flooding: the Gidra river case, Slovakia

**DOI:** 10.1016/j.dib.2025.112321

**Published:** 2025-11-25

**Authors:** Matej Vojtek, Ľubomír Benko, Jozef Kapusta, Dávid Držík, Michal Munk, Daša Munková, Martin Drlík, Jana Vojteková

**Affiliations:** a Department of Geography, Geoinformatics and Regional Development, Faculty of Natural Sciences and Informatics, Constantine the Philosopher University in Nitra, Trieda A. Hlinku 1, 949 01 Nitra, Slovakia; b Institute of Geography, Slovak Academy of Sciences, Štefánikova 49, 814 73 Bratislava, Slovakia; c Department of Informatics, Faculty of Natural Sciences and Informatics, Constantine the Philosopher University in Nitra, Trieda A. Hlinku 1, 949 01 Nitra, Slovakia; d Institute of Security and Computer Science, University of the National Education Commission, Krakow, ul. Podchorążych 2, 30-084 Kraków, Poland; e Science and Research Centre, University of Pardubice, Studentská 84, 532 10 Pardubice, Czech Republic

**Keywords:** Slope, Height above the nearest drainage, Topographic wetness index, Stream power index, Euclidean distance from river, Manning’s roughness, Normalized difference vegetation index

## Abstract

This data article releases geospatial predictor files for a section of the Gidra River (western Slovakia). Based on the last cycle of the Preliminary Flood Risk Assessment from 2024 in Slovakia, the past fluvial floods and, especially, the flash flood from 7 June 2011 affected the studied river section and the Píla municipality significantly. These facts resulted in including the studied Gidra River section to critical river sections for the occurrence of fluvial floods. The collection comprises seven single-band rasters on a common 1 m grid: slope, Topographic Wetness Index, Stream Power Index, Height Above the Nearest Drainage, Euclidean distance from river, surface roughness, and Normalized Difference Vegetation Index. Source inputs are the LiDAR digital elevation model (DMR 5.0, resolution: 1 m) from 2017 and aerial orthophotos (resolution: 0.15 m) from 2023. All layers are georeferenced to a single CRS, co-registered to identical extent and transform, and provided as GeoTIFFs with documented units, no data, and datatypes. Processing relied on ArcGIS 10.2.2 software and Python for alignment and quality checks. Additional tables supply per-file inventory and descriptive statistics (min/mean/max/std) to enable automated validation and integration into geographic information system (GIS) and modelling workflows. The dataset is designed for reuse in flood-related and terrain–vegetation analyses, including feature engineering, benchmarking, and training of machine-learning (ML) models that require uniform, high-resolution predictors.

Specifications Table

**Table:** 

Subject	Earth & Environmental Sciences
Specific subject area	Geospatial predictors for ML-based fluvial flood extent modelling (slope, HAND, TWI, SPI, distance from river, NDVI, surface roughness)
Type of data	Images in TIFF format
Data collection	The data represents a critical section of the Gidra River, which is located in the western part of Slovakia. The primary inputs for creating the physical-geographical predictors were the airborne laser scanned Light Detection and Ranging (LiDAR) digital elevation model (DMR 5.0) with a resolution of 1 m as well as the orthophotos from 2023 (15 cm pixel), provided freely by the Geodetic and Cartographic Institute, Bratislava. The LiDAR DEM was laser-scanned during the non-vegetation period (November 2017–April 2018) while the orthophotos were taken during the vegetation period (May–September 2023). Another specifications of the LiDAR point cloud collection were as follows: last reflection point density: minimum 5 per m^2^, overlay of scanned strips: minimum 20 %, beam trace: 0.25 m, and crossbars: one for each flight. The vertical accuracy of the original LiDAR point cloud is 0.07 m and the positional accuracy is 0.15 m. Rasters of slope, SPI, TWI, and HAND were created based on the LiDAR DEM. Raster of distance from river was created using the Euclidean distance based on the vector layer of river centreline. NDVI raster was created based on the orthophotos from 2023 with positional accuracy of 0.17 m. The NDVI raster was resampled from original resolution of 15 cm to 1 m. Another source data for creating the raster of Manning´s roughness was the Basic Data Base for the Geographic Information System (ZBGIS) from 2023, acquired also from the Geodetic and Cartographic Institute, Bratislava. The ZBGIS is the spatial database of objects and land use/land cover classes, which is annually updated by the Geodetic and Cartographic Institute based on current orthophotos and cadastral maps. The collected data (LiDAR: tiff files and orthophoto: tiff+tfw files) were processed in ArcGIS 10.2.2 software.
Data source location	Study area: Gidra River section, western Slovakia. S-JTSK (JTSK03) / Krovak East North, EPSG:8351; units metres; order E, N: top-left = −555 448, −1 254 175; bottom-right = −553 148, −1 256 276
Data accessibility	Repository name: Mendeley Data Data identification number: doi: 10.17632/bprmy76fdv.1 Direct URL to data: https://doi.org/10.17632/bprmy76fdv.1 The dataset is publicly available in the Mendeley Data repository.
Related research article	None

## 1 Value of the Data

• Seven 1 m raster predictors, including slope, Height Above the Nearest Drainage (HAND), Topographic Wetness Index (TWI), Stream Power Index (SPI), distance from river, Normalized Difference Vegetation Index (NDVI), surface roughness, plus the source LiDAR digital elevation model (DEM) and orthophoto footprint provide a compact, high-resolution stack for fluvial flood modeling applications.
• The uniform grid and per-file statistics make the dataset ready for modelling, benchmarking and method comparison, especially geographic information system (GIS)-based and machine learning (ML) hydrological approaches. The predictors can be used to train flood scenarios for predicting the fluvial flood extent as well as flow depth.
• Policymakers can use this dataset for decision making and design of flood-resilient approaches. It can help better understand how to approach similar terrain layout in flood prediction and prevention.
• The predictors are broadly reusable beyond floods prediction (e.g. erosion potential or geomorphological analysis), supporting cross-domain analysis.

## 2 Background

The dataset compiles a set of single-band raster predictors on a 1 m grid for a section of the Gidra River located in the western Slovakia. The section of the Gidra River represents a 3.1 km critical river section with a bed slope of 0.018 m/m. A 250 m buffer zone with an area of ​​1.5 km^2^ was created around the investigated river section. The altitude ranges from 212 to 371 m above sea level. The stack includes terrain morphology (DEM, slope), flow-related information (TWI, SPI, HAND), hydro-proximity (Euclidean distance from river), surface roughness (Manning’s n values), and vegetation state (NDVI from RGB+NIR orthophotos). Source inputs are the LiDAR DEM (DMR 5.0, resolution: 1 m) and orthophotos (resolution: 0.15 m). Each layer is georeferenced and aligned to the same extent and transform. The accompanying statistics provide file-level metadata to assist reuse and validation. The aim is to provide a reproducible predictor set suitable for training and evaluating flood-related workflows and other terrain analyses. In combination with similar datasets [[1], [2], [3]], this can create a valuable source for flood prediction and prevention.

## 3 Data Description

The dataset comprises of thematic maps for the Gidra River section that is prone to fluvial flooding [4]. The dataset provides raw orthophoto map, LiDAR DEM, and shapefile that were the basis for creating predictors. These predictors can be further used to train flood scenarios for predicting the fluvial flood extent as well as flow depth. Seven raster predictors for fluvial flood extent modelling at 1 m resolution were created from the orthophoto source file and LiDAR DEM (Table 1).

**Table 1:** Dataset file composition.

Name	Description	Pixel area m^2^	Bands	CRS (EPSG)	CRS name
Orthophoto_Gidra.tif	Aerial orthophoto, RGB+NIR	0.0225	4	EPSG:8351	S-JTSK (JTSK03) / Krovak East North
DEM_Gidra.tif	LiDAR Digital Elevation Model (DEM)	1.0	1	EPSG:8351	S-JTSK (JTSK03) / Krovak East North
Shapefile_Gidra.shp	River Centreline Shapefile	-	1	EPSG:8351	S-JTSK (JTSK03) / Krovak East North
HAND_Gidra.tif	Height Above Nearest Drainage (HAND)	1.0	1	EPSG:8351	S-JTSK (JTSK03) / Krovak East North
EucDist_Gidra.tif	Euclidean Distance from river	1.0	1	EPSG:8351	S-JTSK (JTSK03) / Krovak East North
Roughness_Gidra.tif	Surface roughness (Manning´s n values)	1.0	1	EPSG:8351	S-JTSK (JTSK03) / Krovak East North
SPI_Gidra.tif	Stream Power Index (SPI)	1.0	1	EPSG:8351	S-JTSK (JTSK03) / Krovak East North
TWI_Gidra.tif	Topographic Wetness Index (TWI)	1.0	1	EPSG:8351	S-JTSK (JTSK03) / Krovak East North
NDVI_Gidra.tif	Normalized Difference Vegetation Index (NDVI)	1.0	1	EPSG:8351	S-JTSK (JTSK03) / Krovak East North
Slope_Gidra.tif	Slope	1.0	1	EPSG:8351	S-JTSK (JTSK03) / Krovak East North

All predictors are single-band GeoTIFFs at 1 m pixel size on a common grid (2300 × 2101). File-level metadata (CRS, transform, NoData) are stored in the TIFF headers. The data repository [5] has the following structure:

• Orthophoto_Gidra.tif (aerial orthophoto, RGB+NIR),
• DEM_Gidra.tif (elevation grid),
• Shapefile_Gidra.shp (shapefile, LineString geometry),
• HAND_Gidra.tif (Height Above the Nearest Drainage),
• EucDist_Gidra.tif (Euclidean distance from river),
• Roughness_Gidra.tif (Manning’s n roughness),
• SPI_Gidra.tif (Stream Power Index),
• TWI_Gidra.tif (Topographic Wetness Index),
• NDVI_Gidra.tif (Normalized Difference Vegetation Index),
• Slope_Gidra.tif (slope grid).

Descriptive statistics (Table 2) offer a comprehensive insight into each raster to better understand value ranges, variability, and basic distribution shape. The 4-band orthophoto maps shows the expected 8-bit digital number range (0–255) with band means of 58.02 (Red), 84.58 (Green), 79.41 (Blue), and 124.49 (NIR). GIS-based derived predictors include SPI (0–22,882.91), TWI (−1.5–15.93), HAND (0–121.70 m), planar Euclidean distance from river (0–250 m), NDVI (−0.75–1.00), and slope in degrees (0–78.85°). Roughness contains Manning’s n values between 0.02 and 0.15, reflecting its class-based origin. The descriptive analysis of files can offer detection of unit mistakes, no-data leakages, or out-of-range values.

**Table 2:** Descriptive statistics for .tif files.

Name	Min	Max	Mean	Std.Dev
Orthophoto_Gidra.tif (Red)	0.00	255.00	58.02	31.13
Orthophoto_Gidra.tif (Green)	0.00	255.00	84.58	31.45
Orthophoto_Gidra.tif (Blue)	0.00	255.00	79.41	26.63
Orthophoto_Gidra.tif (NIR)	0.00	255.00	124.49	38.23
DEM_Gidra.tif	212.51	370.89	268.19	32.59
HAND_Gidra.tif	0.00	121.70	29.93	25.43
EucDist_Gidra.tif	0.00	250.00	122.68	72.23
Roughness_Gidra.tif	0.02	0.15	0.11	0.05
SPI_Gidra.tif	0.00	22,882.91	15.26	158.86
TWI_Gidra.tif	−1.50	15.93	3.95	1.84
NDVI_Gidra.tif	−0.75	1.00	0.34	0.20
Slope_Gidra.tif	0.00	78.85	15.05	10.67

**Fig. 1:**
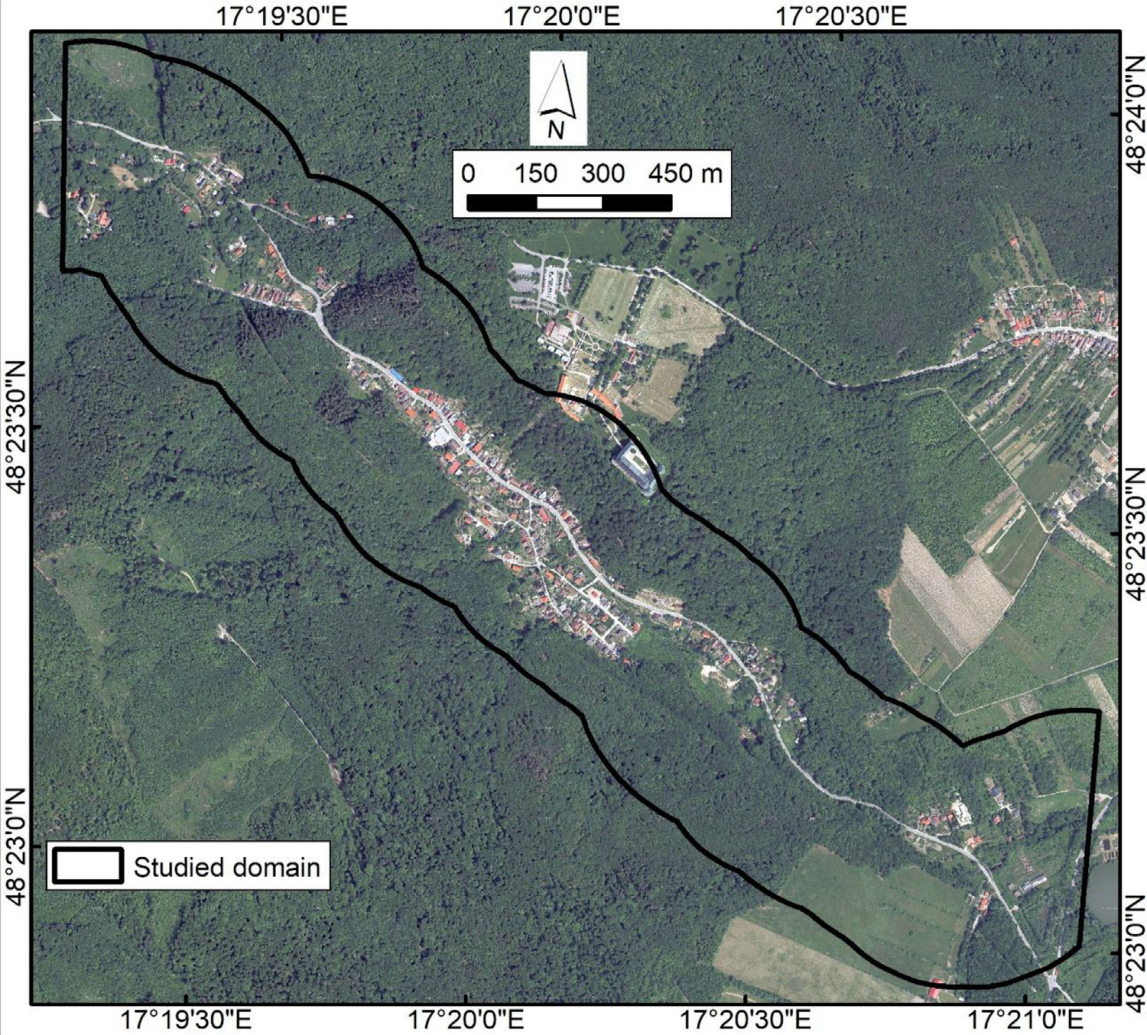
Preview of the orthophoto source file.

The source orthophoto map consists of 4 bands (Red, Green, Blue, NIR), where together with the LiDAR DEM file it offers basis for creating predictors. A down-sampled RGB view of Orthophoto_Gidra.tif is depicted on Fig. 1, covering the same footprint as the 1 m rasters. The source data were obtained for a study area of Gidra River, which is located in the western part of Slovakia. The primary input for creating the morphometric predictors was the airborne laser scanned LiDAR digital elevation model (DMR 5.0) with a resolution of 1 m, provided freely by the Geodetic and Cartographic Institute, Bratislava at the following link: https://opendata.skgeodesy.sk/static/LLS/DMR5/DMR5_0_sjtsk03_bpv.zip.

## 4 Experimental Design, Materials and Methods

Fluvial flood mapping using ML or other approaches require quality data. The dataset is focused on the predictors processed by GIS and remote sensing based methods and suitable for ML analysis. Source RGB+NIR orthophoto from 2023 (https://opendata.skgeodesy.sk/static/Ortofotomozaika_SR/3_cyklus_2023-2025/orto_nova_farba_2023_zapad_rgbn_1.zip) and LiDAR DEM – DMR 5.0 (https://opendata.skgeodesy.sk/static/LLS/DMR5/DMR5_0_sjtsk03_bpv.zip) provided by the Geodetic and Cartographic Institute, Bratislava, were used to derive the predictors that can be used in ML approaches to model fluvial floods. ArcGIS 10.2.2 software was used to produce the predictors based on various approaches and Python 3.9.23 with libraries rasterio 1.4.3, numpy 2.0.2, geopandas 1.0.1 and matplotlib 3.9.4 were used to validate the data.

Seven raster predictors for fluvial flood extent modelling at 1 m resolution were created:

• Height Above the Nearest Drainage (HAND), parameters: rasters of river centerline, filled DEM, flow direction, and flow accumulation,
• Euclidean Distance from River, parameters: vector layer of river centerline,
• Surface Roughness, parameters: raster of land use/land cover, Manning´s n values,
• Stream Power Index (SPI), parameters: rasters of slope and flow accumulation,
• Topographic Wetness Index (TWI), parameters: rasters of slope and flow accumulation,
• Normalized Difference Vegetation Index (NDVI), parameters: near-infrared band and red band of orthophoto,
• Slope, parameters: DEM.

**Fig. 2:**
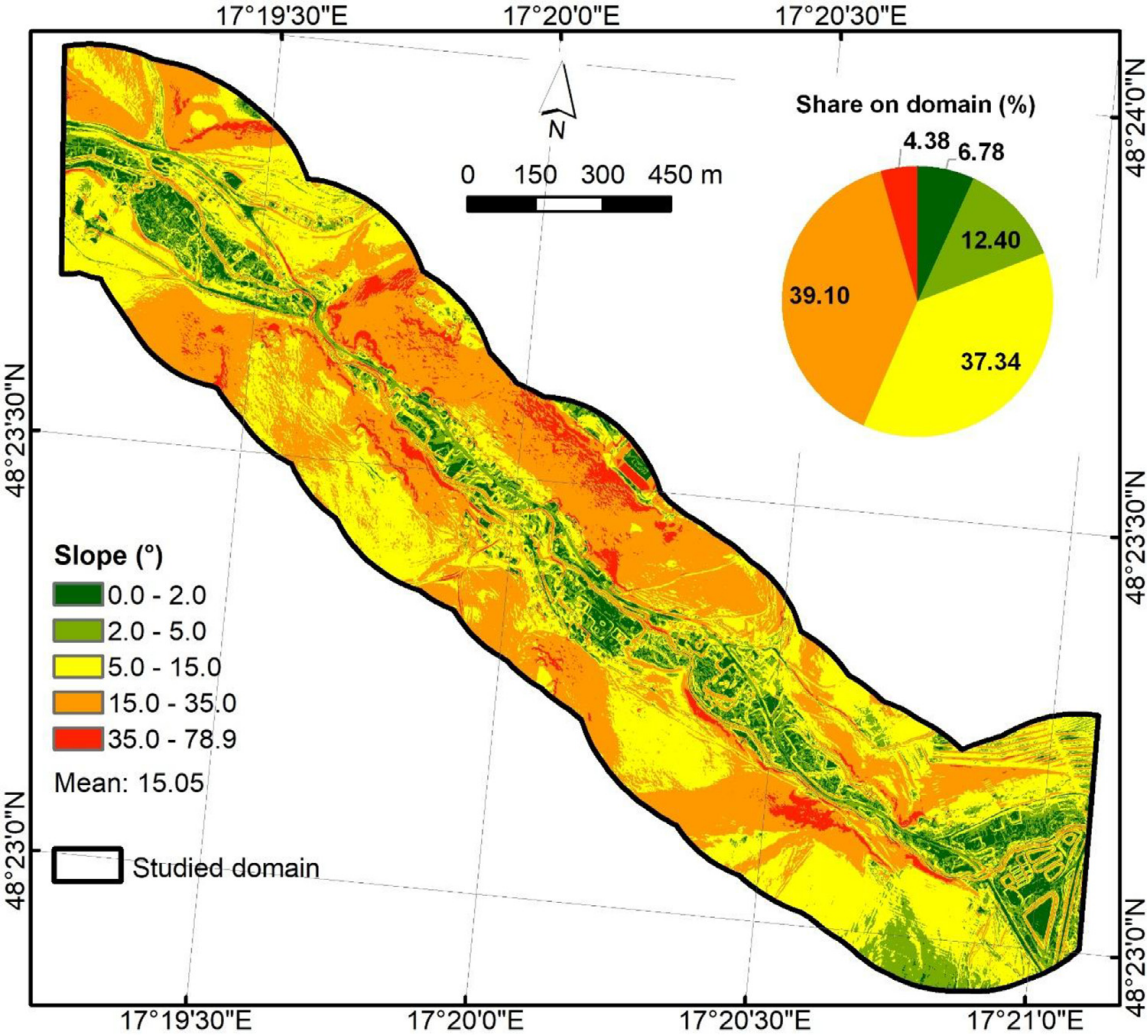
Slope predictor.

The main input in creating these predictors was the airborne laser scanned LiDAR digital elevation model (DMR 5.0) with a resolution of 1 m and orthophotos from 2023 (15 cm pixel), provided by the Geodetic and Cartographic Institute, Bratislava. LiDAR digital elevation model (DEM) was the basis for creating predictors of slope, SPI, TWI, and HAND. The output tiff file was aligned to the reference grid and stored as float32 (NoData = −9999). Slope (Fig. 2) was derived in degrees from the 1 m DEM using the ArcGIS 10.2.2 software and stored as float32 on the 1 m grid (Z-factor = 1).

**Fig. 3:**
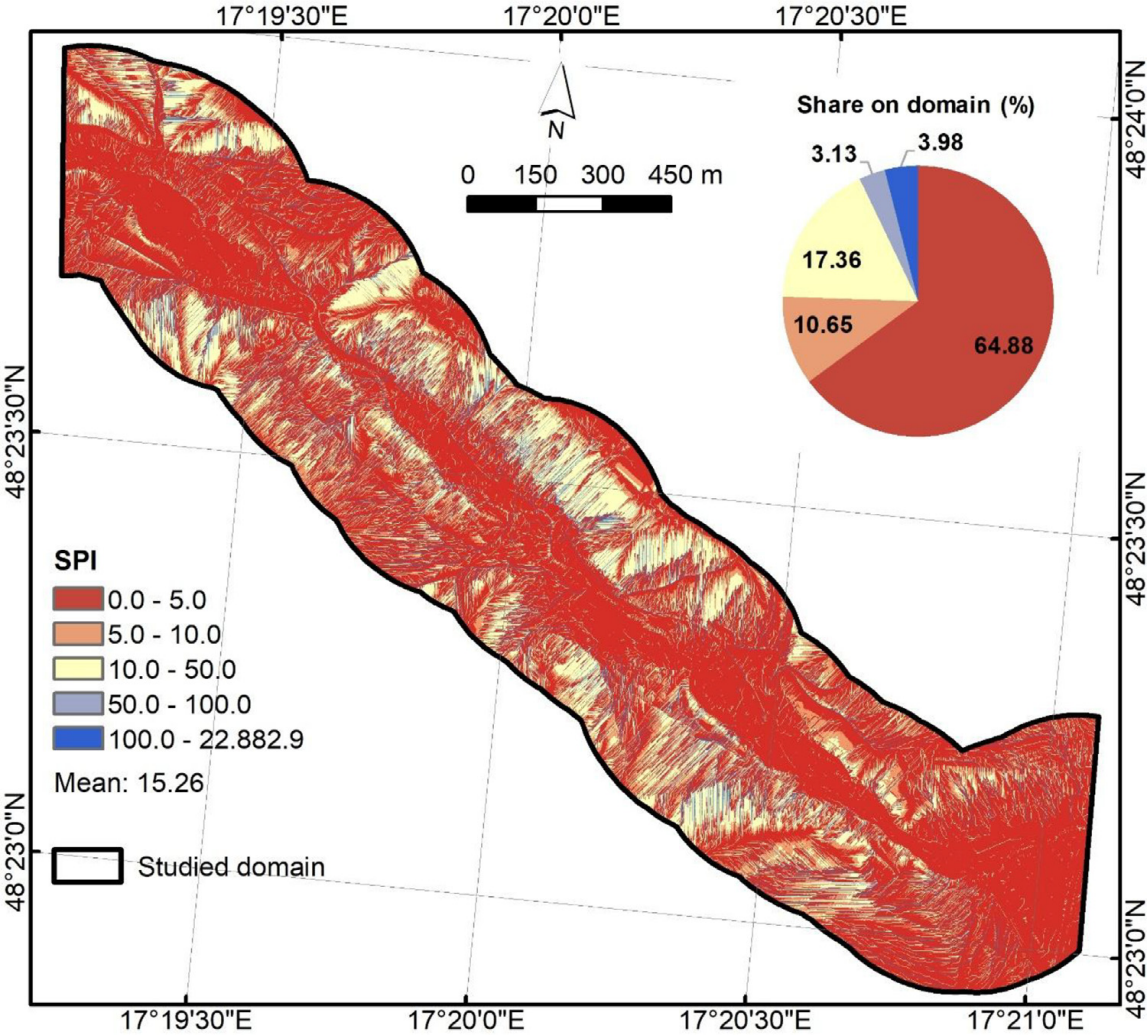
Stream power index predictor.

ArcGIS 10.2.2 software was used to derive the SPI, TWI, and HAND using the following equations. SPI (Fig. 3) was derived as

SPI=ln(A×tanβ),

where A is the local upslope area draining through a certain point per unit contour length and tanβ is the slope gradient in radians. Output was stored as float64 (NoData = −9999) without additional applied scaling.

**Fig. 4:**
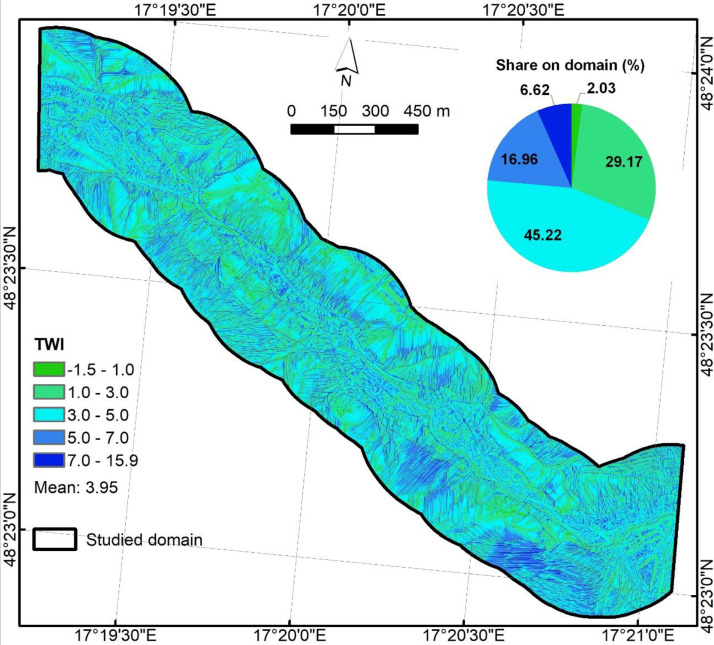
Topographic wetness index predictor.

TWI (Fig. 4) was calculated following

$$TWI=\ln\left(\frac{A}{\tan\beta }\right),$$

using the same parameters A and tanβ. Output was saved as float64 (NoData = −9999).

**Fig. 5:**
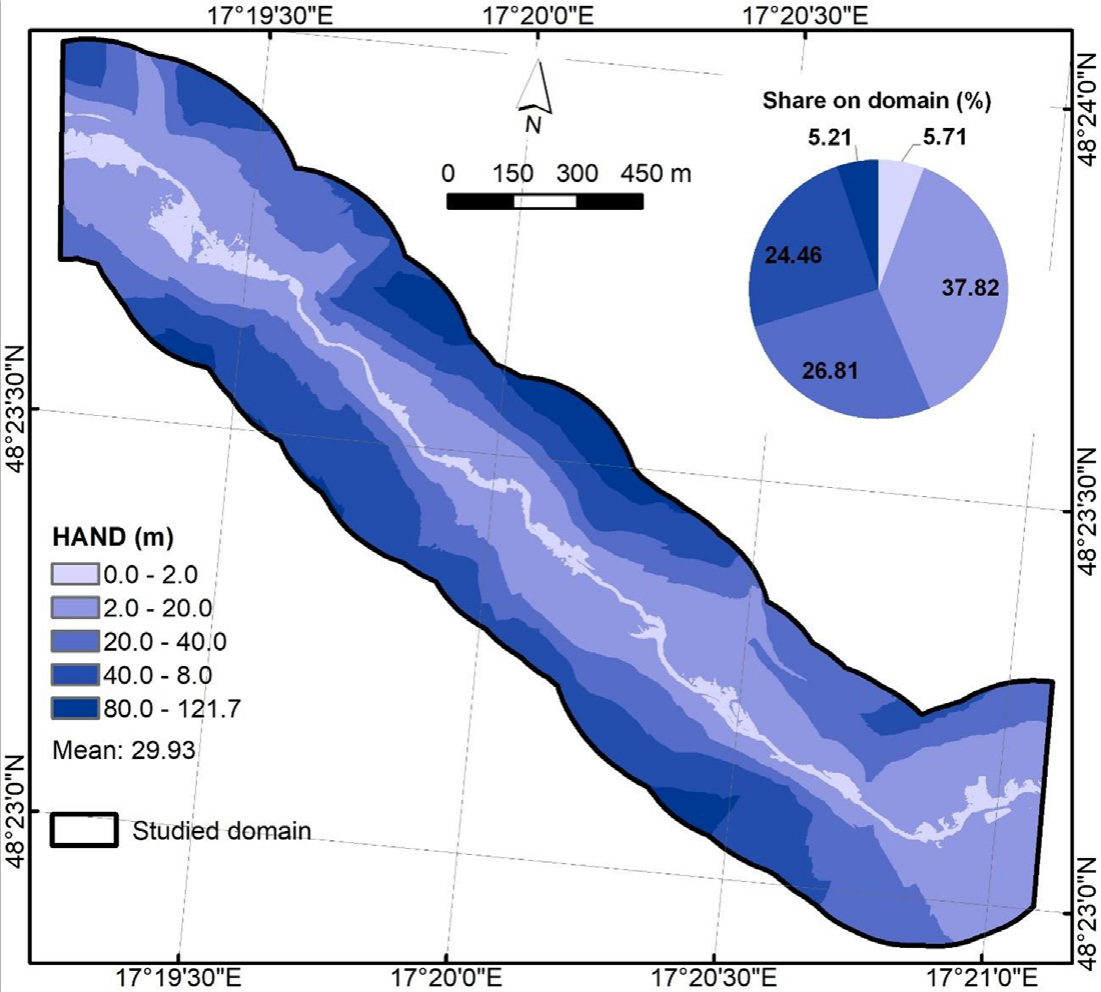
Height above the nearest drainage predictor.

HAND (Fig. 5) was computed using ArcGIS 10.2.2 software following the approach of Nobre et al. [6] and Vojtek et al. [7] with the primary inputs of the raster of the river section, filled DEM, derived flow direction and flow accumulation rasters. Output was stored as float32 (NoData = −9999).

**Fig. 6:**
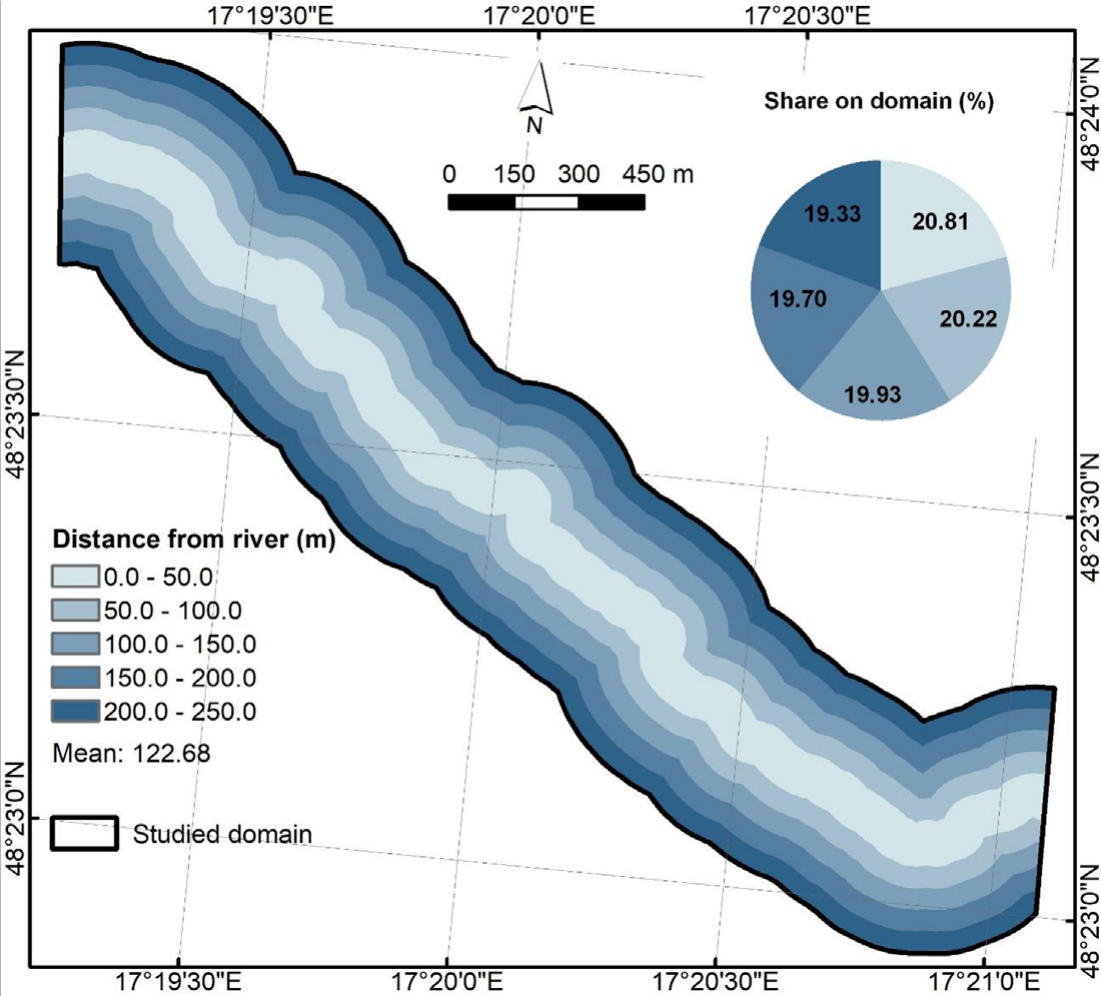
Euclidean distance from river predictor.

Euclidean distance from river (Fig. 6) is represented as minimum planar distance from each cell to the river centerline of the studied river reach. Output was stored as float32 (NoData = −9999).

**Fig. 7:**
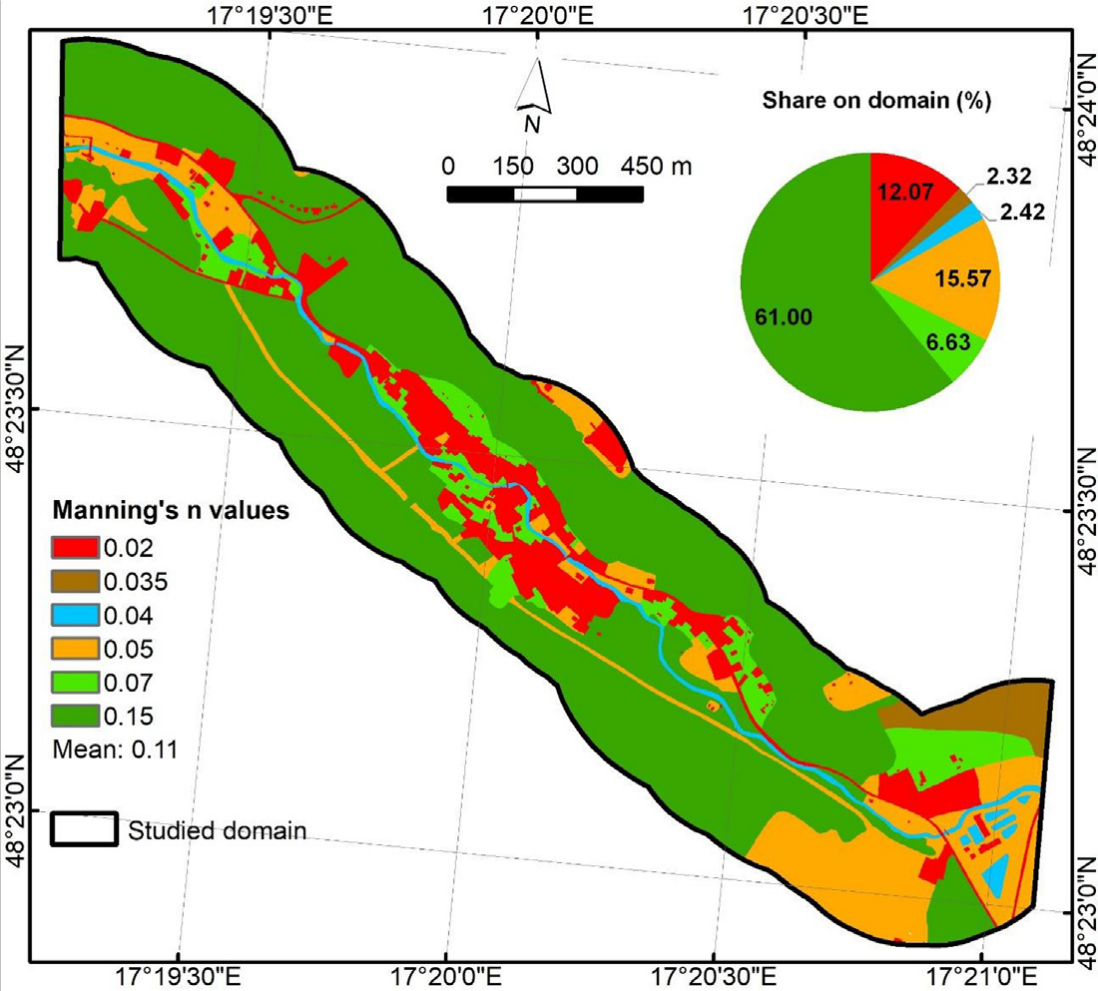
Surface roughness predictor.

Surface roughness (Fig. 7) was assigned from the Basic Data Base for the Geographic Information System (ZBGIS) from 2023. The created land use/land cover (LULC) 1 m raster with the output stored as float with discrete values. ZBGIS 2023 raster dataset is openly provided by the Geodetic and Cartographic Institute, Bratislava at the following link: https://opendata.skgeodesy.sk/static/ZBGIS_Raster/zbgis_raster_5000_2023.zip. Manning’s roughness coefficients were assigned to 11 LULC classes, as follows [8]:

• Built-up area (0.02),
• Road (0.02),
• Railway (0.02),
• Urban greenery (0.05),
• Forest (0.15),
• Grassland (0.05),
• Material/waste dump (0.03),
• Orchard/garden (0.07),
• Arable land (0.035),
• Channel (0.04),
• Water body (0.04).

**Fig. 8:**
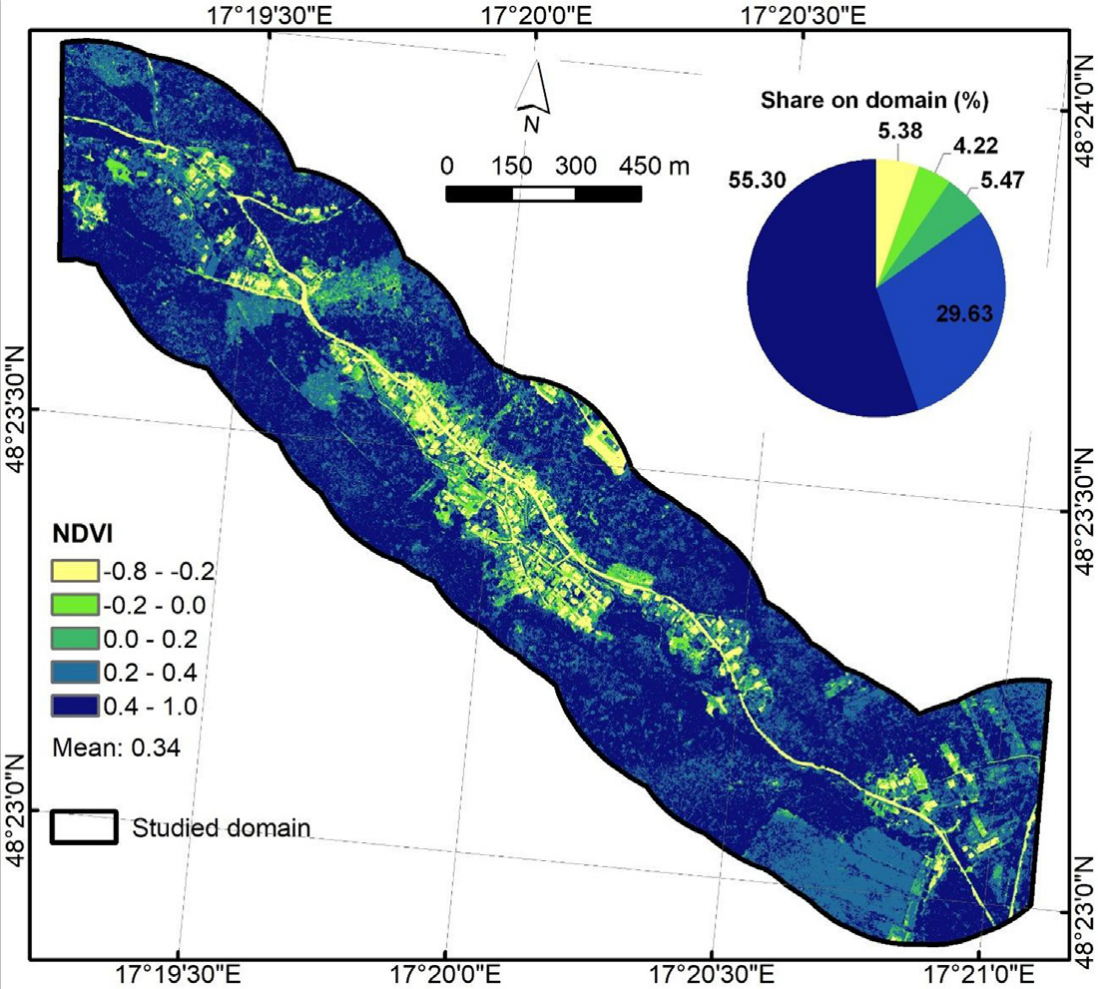
Normalized difference vegetation index predictor.

NDVI (Fig. 8) was created from the orthophotos from 2023 with 15 cm resolution, provided by the Geodetic and Cartographic Institute, Bratislava using the following equation:

$$NDVI=\frac{NIR-RED}{NIR+RED},$$

where NIR is the reflectance in the near-infrared band and RED is reflectance in the red band of aerial imagery. The NDVI raster with 15 cm resolution was resampled to 1 m to match the LiDAR DEM-based predictors and stored as float32.

## Limitations

The main limitation is the region-specific dataset of a single river section, so geographic transferability and generalization was not tested. The dataset is a static snapshot without time series of data. As for the original LiDAR point cloud, the vertical accuracy is 0.07 m and the positional accuracy is 0.15 m. The positional accuracy of the orthophotos is 0.17 m. Another limitation is in resolution disharmony between the source LiDAR DEM (1 m pixel) and orthophotos (15 cm pixel). During the resampling of the NDVI predictor from 15 cm to 1 m pixel, we lose the original pixel value. Moreover, there is also a temporal disharmony between these two sources, as the LiDAR DEM was laser-scanned during November 2017–April 2018 while the orthophotos were taken during the vegetation period (May–September) of 2023. However, it needs to be noted that the second cycle of the new LiDAR DEM should be available for individual LOT sites between 2022 and 2026. For the studied section of Gidra River, the new LiDAR DEM was not processed so far. The revised LiDAR-derived predictors can be then created for the new time frame following the same procedure described in this study.

## Ethics Statement

The authors have read and follow ethical requirements for publication in Data in Brief. The authors confirm that the current work does not involve human subjects, animal experiments, or any data collected from social media platforms.

## CRediT Author Statement

**Matej Vojtek:** Conceptualization, Methodology, Investigation, Software, Data curation, Formal analysis, Writing – Original draft preparation, Writing – Reviewing and editing, Supervision, Project administration, Funding acquisition. **Ľubomír Benko:** Methodology, Software, Investigation, Data Curation, Writing - Original Draft, Visualization. **Jozef Kapusta:** Methodology, Software, Investigation, Data Curation, Writing - Review & Editing, Visualization. **Dávid Držík:** Methodology, Software, Investigation, Data Curation, Writing - Review & Editing, Visualization. **Michal Munk:** Validation, Formal analysis, Resources, Writing - Review & Editing, Supervision. **Daša Munková:** Validation, Formal analysis, Resources, Writing - Review & Editing. **Martin Drlík:** Validation, Formal analysis, Resources, Data Curation, Writing - Review & Editing, Supervision. **Jana Vojteková:** Methodology, Resources, Software, Data curation, Writing - Review & Editing, Visualization.

## Data Availability

Mendeley DataDataset of physical-geographical predictors (Original data).
